# Patterns of treatment with everolimus exemestane in hormone receptor-positive HER2-negative metastatic breast cancer in the era of targeted therapy

**DOI:** 10.1186/s13058-021-01394-y

**Published:** 2021-01-29

**Authors:** Mariya Rozenblit, Sophia Mun, Pamela Soulos, Kerin Adelson, Lajos Pusztai, Sarah Mougalian

**Affiliations:** 1 Yale School of Medicine, 333 Cedar St., PO Box 208032, New Haven, CT 05620 USA; 2 Yale COPPER, Harkness Office Building, 367 Cedar Street, New Haven, CT 06510 USA

**Keywords:** Everolimus, Exemestane, CDK4/6 inhibitors, Metastatic hormone-positive HER2-negative breast cancer, Sequence of therapy, Endocrine therapy, Targeted therapy

## Abstract

**Background:**

There is currently no clinical trial data regarding the efficacy of everolimus exemestane (EE) following prior treatment with CDK4/6 inhibitors (CDK4/6i). This study assesses the use and efficacy of everolimus exemestane in patients with metastatic HR+ HER2− breast cancer previously treated with endocrine therapy (ET) or endocrine therapy + CDK4/6i.

**Methods:**

Retrospective analysis of electronic health record-derived data for HR+ HER2− metastatic breast cancer from 2012 to 2018. The proportion of patients receiving EE first-line, second-line, or third-line, and the median duration of EE prior to next line of treatment (TTNT) by line of therapy was calculated. OS for patients receiving EE first-line, second-line, or third-line, indexed to the date of first-line therapy initiation and stratified by prior treatment received, was calculated with Kaplan-Meier method with multivariable Cox proportional hazards regression analysis.

**Results:**

Six hundred twenty-two patients received EE first-line (*n* = 104, 16.7%), second-line (*n* = 273, 43.9%) or third-line (*n* = 245, 39.4%). Median TTNT was 8.3 months, 5.5 months, and 4.8 months respectively. Median TTNT of EE second-line was longer following prior ET alone compared to prior ET + CDK4/6i (6.2 months (95% CI 5.2, 7.3) vs 4.3 months (95% CI 3.2, 5.7) respectively, *p* = 0.03). Similarly, EE third-line following ET alone vs ET + CDK4/6i in first- or second-line resulted in median TTNT 5.6 months (95% CI 4.4, 6.9) vs 4.1 months (95% CI 3.6, 6.1) respectively, although this was not statistically significant (*p* = 0.08). Median OS was longer for patients who received EE following prior ET + CDK4/6i. EE second-line following ET + CDK 4/6i vs ET alone resulted in median OS 37.7 months vs. 32.7 months (*p* = 0.449). EE third-line following ET + CDK4/6i vs prior ET alone resulted in median OS 59.2 months vs. 40.8 months (*p* < 0.010). This difference in OS was not statistically significant when indexed to the start of EE therapy.

**Conclusion:**

This study suggests that EE remains an effective treatment option after prior ET or ET + CDK4/6i use. Median TTNT of EE was longer for patients who received prior ET, whereas median OS was longer for patients who received prior ET + CDK4/6i. However, this improvement in OS was not statistically significant when indexed to the start of EE therapy suggesting that OS benefit is primarily driven by prior CDK4/6i use. EE remains an effective treatment option regardless of prior treatment option.

**Supplementary Information:**

The online version contains supplementary material available at 10.1186/s13058-021-01394-y.

## Background

Breast cancer remains the most common cancer and the second most common cause of cancer death in women. Metastatic breast cancer is not curable, and the overall survival (OS) at 5 years is 27% [1]. Approximately 75% of breast cancers are hormone receptor positive and human epidermal growth factor receptor 2 negative (HER2−) [2]. For hormone receptor-positive metastatic breast cancer, endocrine therapy (ET) alone or in combination with a CDK4/6 inhibitor (CDK4/6i) is currently the most common first-line therapy. Endocrine therapy includes strategies to deplete estrogen with aromatase inhibitors (AIs), selective estrogen receptor modulators such as tamoxifen, or downregulation of the estrogen receptor with fulvestrant. However, endocrine therapy resistance eventually occurs, leading to the need for more novel agents or cytotoxic chemotherapy.

The addition of CDK4/6i to endocrine therapy has shown improved PFS and OS in the majority of randomized phase III studies, leading to the approval of endocrine therapy + CDK4/6i as first-line treatment for metastatic hormone-positive breast cancer in 2015 [3–12]. Everolimus in combination with exemestane (EE) was approved in 2012 as therapy for patients with metastatic breast cancer who previously progressed on an AI based on the BOLERO-02 trial. The study showed that progression-free survival was significantly longer with everolimus exemestane compared to exemestane and placebo (7.8 vs 3.2 months) [13]. While an improvement in overall survival of 4.4 months was seen, this was not statistically significant. Subsequent studies have confirmed an improvement in PFS with everolimus and exemestane [14–16].

There is currently no published data regarding the efficacy of everolimus exemestane following treatment with CDK4/6 inhibitors. In this study, we use real-world evidence to assess the use and effectiveness of everolimus exemestane as first-line, second-line, or third-line therapy following prior endocrine therapy alone vs endocrine therapy + CDK4/6i in patients with metastatic hormone-positive HER2− breast cancer.

## Methods

This is a population-based retrospective analysis using electronic health record-derived data collected for women diagnosed with hormone receptor-positive HER2-negative metastatic breast cancer from 2012 to 2018. This time period was selected because everolimus was approved by the US Food and Drug Administration to be used with exemestane in the treatment of postmenopausal women with advanced hormone receptor-positive, HER2-negative breast cancer refractory to treatment with either letrozole or anastrozole in July 2012. The specific aims were to examine (1) the proportion of patients receiving everolimus exemestane as first-line, second-line, or third-line therapy over time; (2) the duration of treatment of everolimus exemestane (defined as time prior to next line of treatment, TTNT) in first-line, second-line, or third-line treatment, stratified by prior lines of therapy; and (3) the overall survival of patients receiving everolimus and exemestane as first-line, second-line, or third-line therapy, stratified by prior lines of therapy and adjusted for covariates.

### Data source

The Flatiron Health database is a nationwide longitudinal, demographically and geographically diverse de-identified database derived from electronic health record (EHR) data curated via technology-enable abstraction. The database includes data from over 280 cancer clinics (~ 800 sites of care) representing more than 2.2 million US cancer patients available for analysis. Institutional Review Board approval of the study protocol was obtained prior to study conduct and included a waiver of informed consent. The Yale Human Investigation Committee determined that this study did not involve human subjects.

### Study sample

**Fig. 1:**
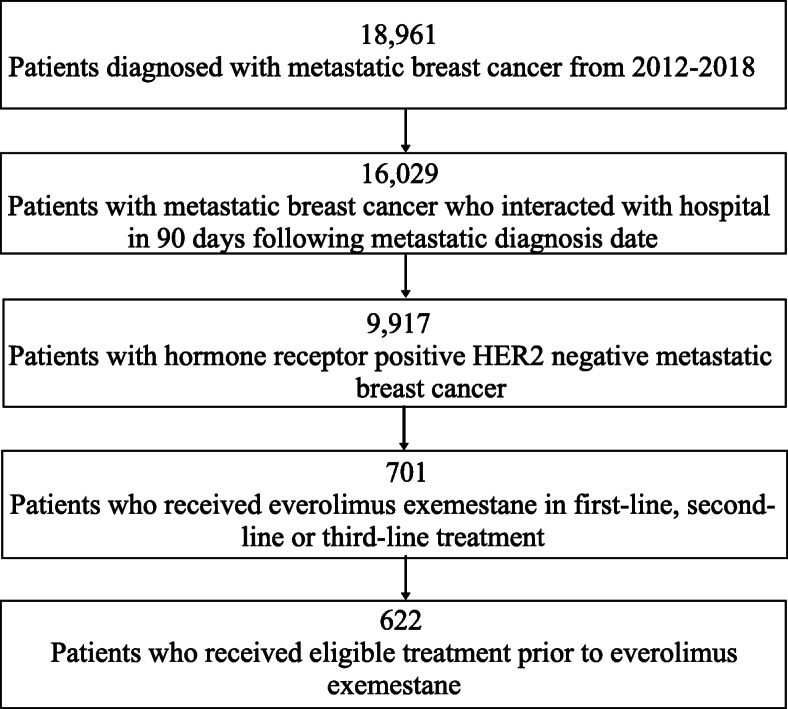
Study population: patients diagnosed with hormone receptor-positive HER2-negative metastatic breast cancer from 2012 to 2018 who received everolimus exemestane in first-line, second-line, or third-line

Cohort eligibility criteria included (1) having hormone receptor-positive HER2-negative breast cancer at the time of metastatic diagnosis and (2) receiving everolimus exemestane in first-line, second-line, or third-line treatment (Fig. 1). For ER, PR, and HER2, all available biomarker test results were collected starting at the initial metastatic diagnosis. Each patient had an International Classification of Diseases 9th or 10th revision (ICD-9 or ICD-10) diagnosis code for breast cancer, had at least two documented visits in the Flatiron Health network on or after January 1, 2012, and had evidence of stage IV or recurrent metastatic breast cancer with a metastatic diagnosis date on or after January 1, 2012, based on confirmation of EHR documents. Patients for whom HER2 was positive, equivocal, or unknown were excluded. Per Flatiron Health’s guidelines, and patients without evidence of a healthcare interaction within 90 days following their metastatic diagnosis date were also excluded.

### Construction of variables

#### Treatment

First-line, second-line, and third-line treatment regimens were categorized into the following four distinct treatment groups: (1) everolimus exemestane (EE), (2) endocrine therapy (ET) alone (including AIs, fulvestrant, or tamoxifen), (3) ET + CDK 4/6i, or (4) chemotherapy (Supplementary Table 1). The proportion of patients receiving everolimus exemestane as first-line, second-line, or third-line therapy was calculated from the entire cohort of patients with hormone receptor-positive HER2-negative metastatic breast cancer from 2012 to 2018. Duration of everolimus exemestane therapy was defined as time to next treatment (TTNT) and was calculated by subtracting the therapy start date from the therapy end date. The therapy end date was defined as the day prior to the start of the next line of treatment. Prolonged TTNT was defined as 6 months or greater.

#### Overall survival

Among decedent patients, overall survival was calculated by subtracting the date of first-line therapy initiation from the date of death [17]. Because Flatiron Health only provides month and year of death to protect patient privacy, date of death was artificially assigned to the middle of the month (i.e., the 15th) for all patients. Among patients who were otherwise censored, overall survival was calculated by subtracting the date of first-line therapy initiation from the date of any last activity (i.e., vital sign collection, lab test, treatment).

#### Covariates of interest

Covariates of interest included patient age (age ≥ 60 years or age < 60 years), gender (male or female), race/ethnicity (Asian, Black, Hispanic, White, or others), ECOG performance status (0, 1, 2, 3, or missing, using the closest ECOG score to the date of metastatic diagnosis), de novo metastatic disease status (i.e., if breast cancer diagnosis date was equivalent to the metastatic diagnosis date), Elixhauser comorbidity score (0, 1, or 2+ using diagnosis codes), practice type (academic or community), location (Northeast, South, Midwest, or West), insurance type (Medicare, Commercial, other [including Medicaid], or uninsured/unknown), sites of metastasis (visceral or non-visceral), and BMI (≤ 30 or > 30). When measuring comorbidity, ICD-9 and ICD-10 diagnosis codes were identified up to 6 months prior to the patient’s date of metastatic diagnosis for comorbidity categories outlined by Elixhauser et al. [18]

### Statistical analysis

Descriptive statistics were used to calculate the proportion of patients receiving everolimus exemestane in first-line, second-line, and third-line treatment over time. Descriptive statistics were also calculated for each independent variable, stratified by everolimus exemestane line of therapy. Time to next treatment (median, in months [95% CI]) was calculated by line of therapy and stratified by previous line of therapy.

**Fig. 2:**
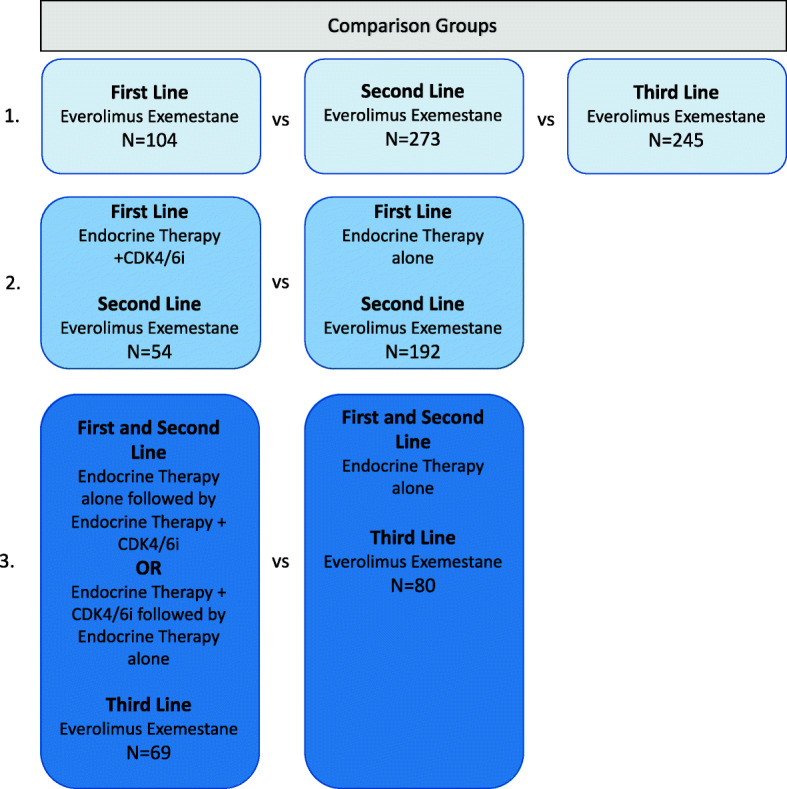
Treatment comparison groups for analyzing the duration of therapy and overall survival

The Kaplan-Meier method was used to compare overall survival for patients receiving everolimus exemestane by line of therapy. Multivariable Cox proportional hazards regression analysis was conducted to estimate hazard ratios (HRs) and 95% confidence intervals (CIs) for patients receiving second- and third-line everolimus exemestane to determine predictor variables associated with survival. For patients receiving second-line everolimus exemestane, patients who received endocrine therapy alone first-line versus endocrine therapy + CDK 4/6i therapy first-line were compared. These comparison groups were chosen since endocrine therapy is the standard first-line treatment for hormone-positive breast cancer and endocrine therapy + CDK4/6i became first-line treatment for this group of patients after 2015. For patients receiving third-line everolimus exemestane, patients who received endocrine therapy alone first-line and second-line versus those who received endocrine therapy + CDK 4/6i first-line or second-line were compared (Fig. 2). For all survival analyses, the date of first-line therapy initiation was considered time zero; however, as a sensitivity analysis, we replicated the analyses using the start of therapy with everolimus exemestane. This was done to assess whether the survival data was mostly driven by everolimus exemestane or prior therapy.

For all models and covariates, it was verified that the proportional hazards assumption was not violated. The following pre-specified covariates were included: prior treatment received (endocrine therapy alone vs endocrine therapy + CDK4/6i), de novo metastatic disease, age at diagnosis, race, ECOG performance status score, comorbidity, sites of metastasis, location, and insurance type. All significance tests were two-sided with an *α* level of 0.05. Analyses were conducted using SAS version 9.4 (SAS Institute, Cary, NC).

## Results

### Baseline characteristics

The final cohort included 622 patients with metastatic breast cancer who received everolimus exemestane (EE) as their first, second, or third line of treatment. The median age was 64 years old; most patients were female (99.2%), were white (69.5%), and had no non-cancer comorbidities prior to the date of metastatic breast cancer diagnosis (92.0%). Most patients received everolimus exemestane as second-line (*n* = 273, 43.9%) or third-line (*n* = 245, 39.4%) treatment compared to first-line (*n* = 104, 16.7%) treatment (Table 1).

**Table 1:**
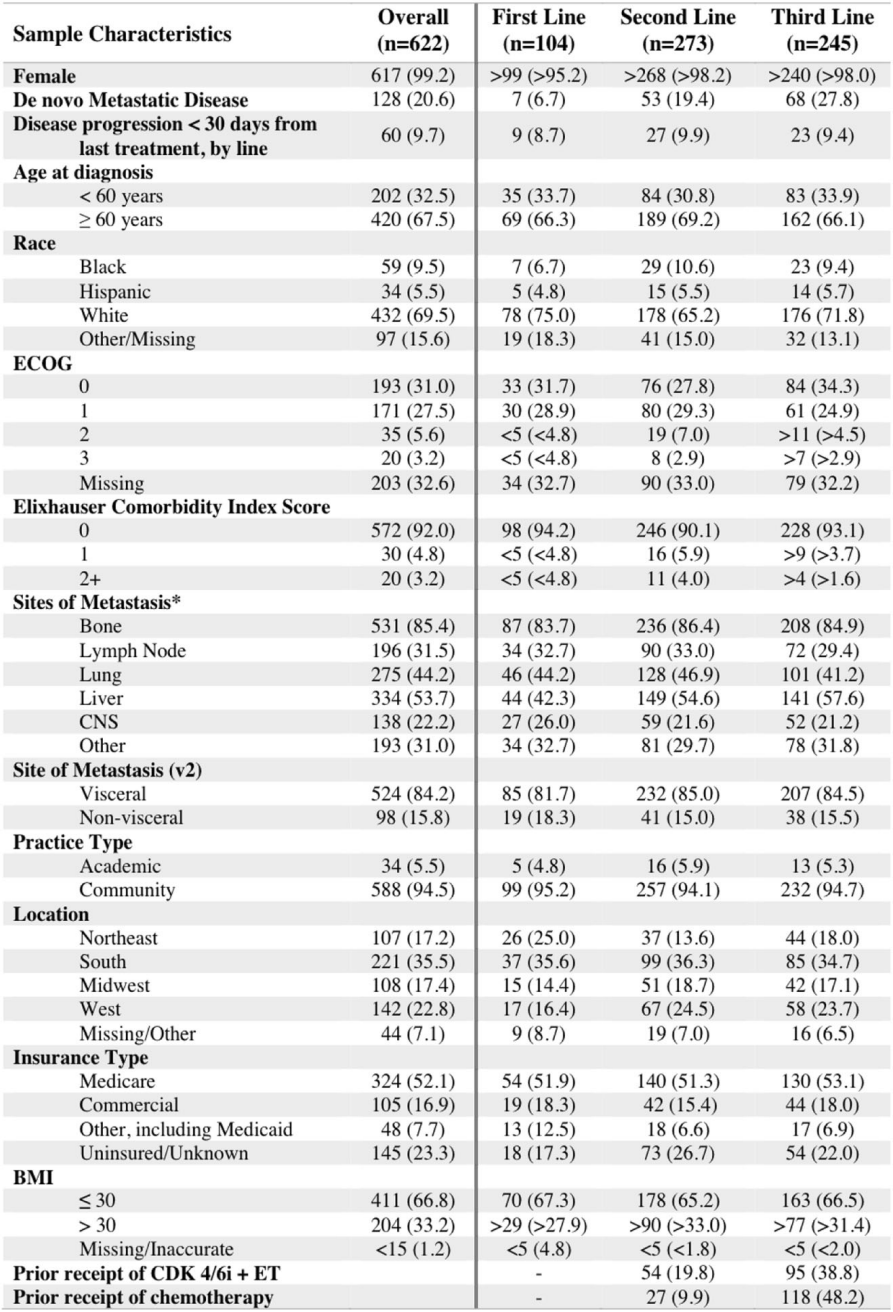
Baseline characteristics for patients with metastatic breast cancer receiving everolimus exemestane in first-, second-, and/or third-line of treatment in the Flatiron Health database, 2012–2018

*Numbers are obscured to protect patient privacy

*Not mutually exclusive; sum > *n* for each group

**Fig. 3:**
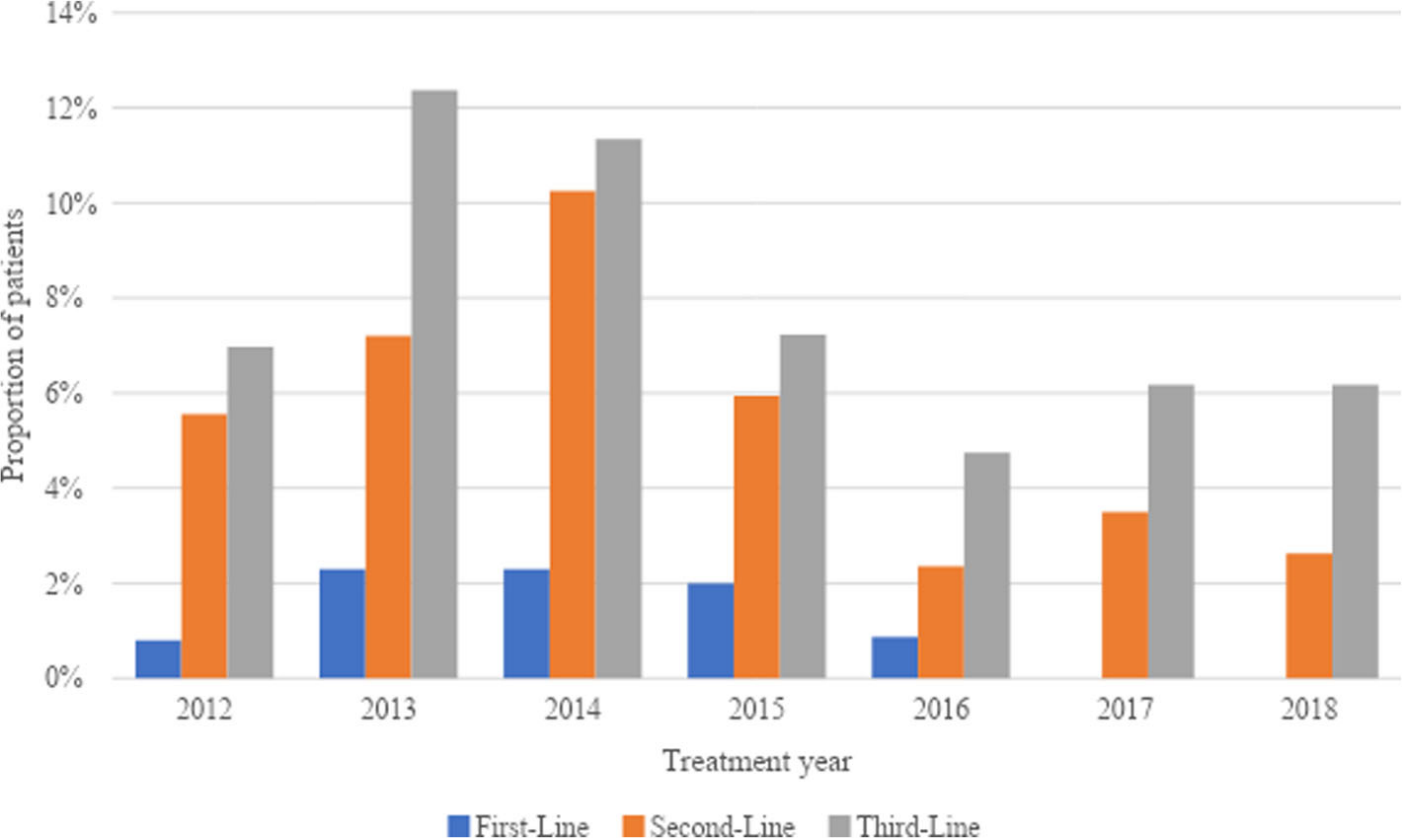
Proportion of patients with hormone receptor-positive HER2-negative metastatic breast cancer receiving everolimus exemestane as first-, second-, and third-line treatment from 2012 to 2018

The proportion of patients receiving everolimus exemestane as first-line therapy increased from 2012 to 2014. There were no patients who received this treatment as first-line therapy from 2017 onwards (Fig. 3). Similar increasing trends were observed for patients receiving everolimus exemestane as second- and third-line therapy from 2012 to 2014, but proportions declined in 2015 and remained relatively stable from 2017 to 2018.

### Time to next treatment (TTNT)

Median TTNT was longer among patients receiving everolimus exemestane as first-line (8.3 months [95% CI 6.0, 11.0]) compared to second-line (5.5 months [95% CI 4.7, 6.3]) and third-line (4.8 months [95% CI 4.2, 5.9]) treatment. More than half of patients who received everolimus exemestane as first-line therapy had a prolonged TTNT (greater than 6 months).

Patients who received endocrine therapy alone prior to everolimus exemestane were more likely to experience a longer TTNT compared to patients who received endocrine therapy + CDK 4/6i. Among patients who received everolimus exemestane as second-line therapy, longer median TTNT was seen when it followed endocrine therapy alone first-line (6.2 months, 95% CI 5.2, 7.3; 52% of patients had prolonged TTNT), vs endocrine therapy + CDK 4/6i first-line (4.3 months, 95% CI 3.2, 5.7; 30% of patients had prolonged TTNT) (*p* = 0.03) (Supplementary Fig. 1a). Similar results were seen in the third-line setting. Among patients who received everolimus exemestane as third-line treatment, longer median TTNT was seen when it followed endocrine therapy alone in first- and second-line treatment (5.6 months, 95% CI 4.4, 6.9; 45% with prolonged TTNT) compared to endocrine therapy + CDK4/6i in first-line or second-line treatment (4.1 months, 95% CI 3.6, 6.1; 38% with prolonged TTNT); however, this difference in median TTNT was not statistically significant (*p* = 0.08) (Supplementary Fig. 1b).

### Overall survival

**Fig. 4:**
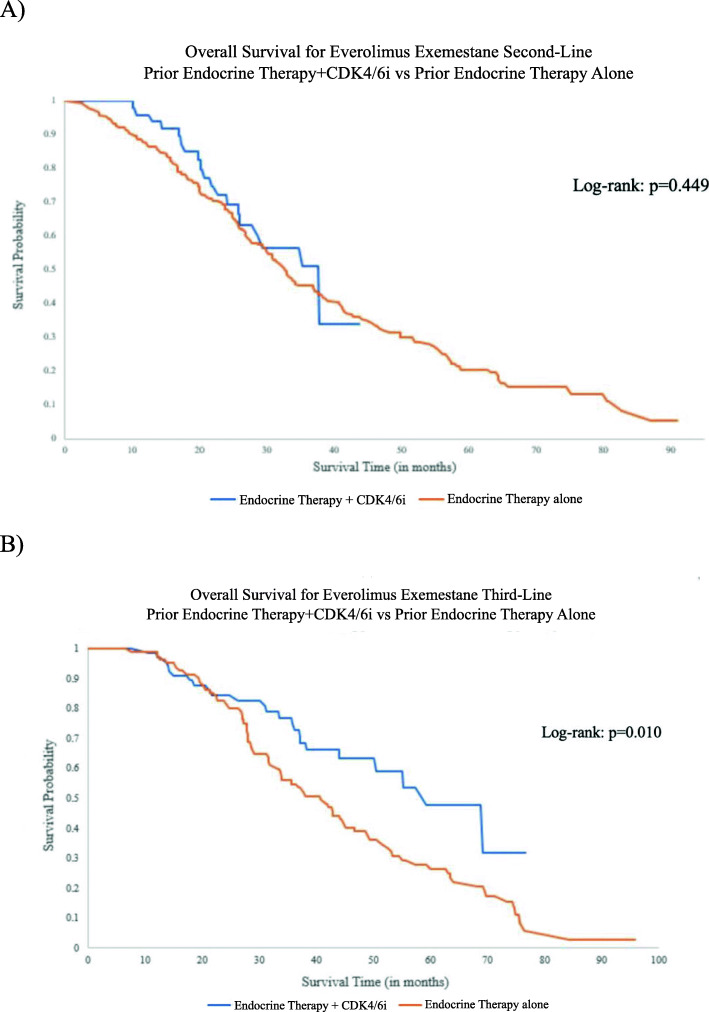
**a** Kaplan-Meier curves for patients receiving everolimus exemestane as second-line treatment, stratified by prior therapy. **b** Kaplan-Meier curves for patients receiving everolimus and exemestane as third-line treatment, stratified by prior therapy

**Fig. 5:**
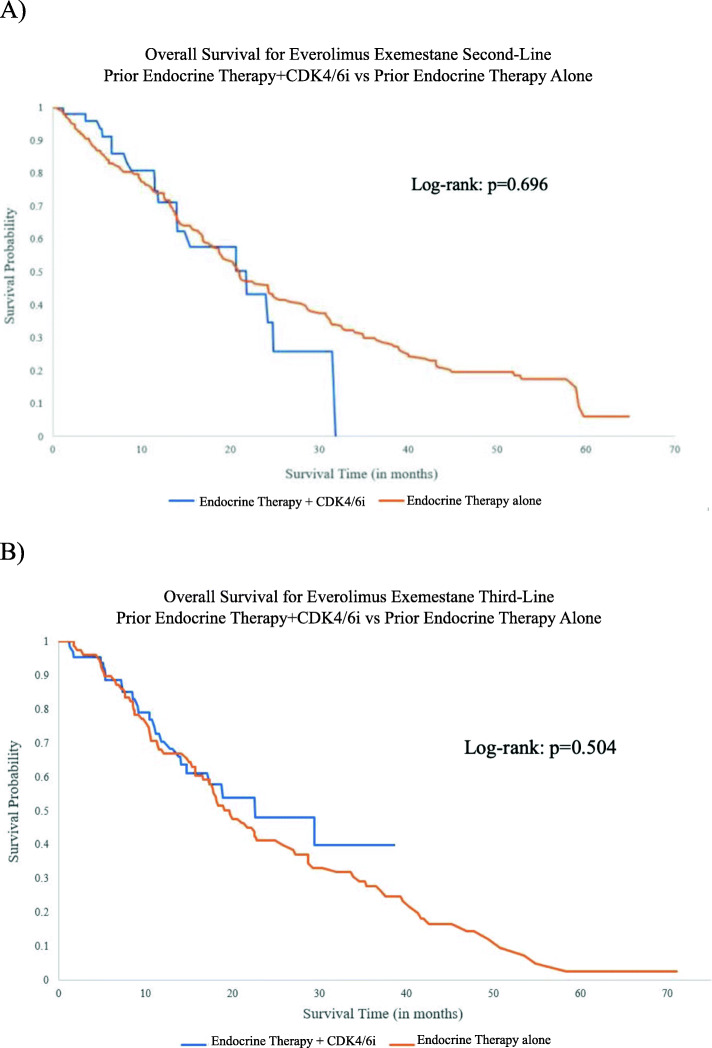
Overall survival calculated using the date of start of everolimus exemestane treatment: **a** Kaplan-Meier curves for patients receiving everolimus exemestane as second-line treatment, stratified by prior therapy; **b** Kaplan-Meier curves for patients receiving everolimus exemestane as third-line treatment, stratified by prior therapy

In our overall cohort of patients who received everolimus exemestane, median overall survival time was longer for patients who received everolimus exemestane as third-line treatment, compared with patients who received it as second- or first-line treatment (40.9 months, 34.0 months, and 34.9 months, respectively). Among patients who received everolimus exemestane as second-line treatment, improved overall survival was seen when it followed endocrine therapy + CDK 4/6i first-line (median OS 37.7 months) compared to endocrine therapy alone first-line (median OS 32.7 months), although this difference was not statistically significant (log-rank *p* = 0.449) (Fig. 4a). Among patients who received everolimus exemestane as third-line treatment, improved overall survival was seen with prior endocrine therapy + CDK 4/6i as first- or second-line treatment (median OS 59.2 months) compared to prior endocrine therapy alone (median OS 40.8 months; log-rank *p* = 0.010) (Fig. 4b, Supplementary Table 2). To investigate whether it was the previous therapy that was driving the overall survival benefit, OS was indexed from the start of everolimus exemestane treatment (rather than the date of first-line therapy initiation). There was no statistically significant difference according to prior treatment received when everolimus exemestane was used as second-line (21.8 months with prior endocrine therapy + CDK4/6i vs 21.0 months with prior endocrine therapy alone) or third-line treatment (22.6 months with prior endocrine therapy + CDK4/6i vs 19.7 months with endocrine therapy alone) (Fig. 5). Hazard ratios and 95% CI’s associated with survival are presented in Supplementary Table 2.

## Discussion

This study suggests that everolimus exemestane remains an effective treatment option after prior CDK4/6i use. Everolimus exemestane in the third-line setting following a combination of prior endocrine therapy + CDK4/6i resulted in a median overall survival of 59.2 months compared to 40.8 months without prior CDK4/6i use (Fig. 4). This is most likely due to the overall survival benefit seen with prior CDK4/6i use. When overall survival was indexed from the start of everolimus exemestane treatment (rather than the date of first-line therapy initiation), the overall survival was similar when everolimus exemestane was used either as second-line or third-line treatment regardless of prior therapy used (Fig. 5). This suggests that the prior use of CDK4/6 inhibitors may be the main driver of improved overall survival in this patient population and also indicates similar efficacy for everolimus exemestane with or without prior CDK4/6 inhibitor therapy.

Interestingly, the time to next treatment (TTNT) with everolimus exemestane was significantly longer when it followed prior endocrine therapy alone vs endocrine therapy + CDK4/6i, regardless of use as second- or third-line treatment. It is unclear why the TTNT of everolimus exemestane was shorter following endocrine therapy + CDK4/6i compared to endocrine therapy alone. This may be due to side effects or decreased tolerability of everolimus exemestane following prior CDK4/6i use; unfortunately, we do not have information regarding side effects, adverse events, reason for discontinuation of therapy, dosage adjustments, or possible mitigation strategies used in our data set. As expected, the TTNT was longest in the patient group receiving everolimus exemestane as first-line therapy compared to second- or third-line therapy, which is most likely due to these patients being treatment-naïve with less resistant disease early in the course of their treatment, and is consistent with data showing longer PFS when everolimus plus an AI was used as first- line vs second-line therapy (22.0 vs 3.7 months) [19].

In our study, the median TTNT of everolimus exemestane second-line following endocrine therapy (6.2 months) was lower than the PFS seen in the BOLERO-02 trial (7.8 months) [14]. This lower TTNT may be due to dose reductions that are frequently necessary when patients develop mucositis, a common side effect of everolimus. Unfortunately, our data set did not include information regarding dosage adjustments for everolimus. However, overall, significant clinical benefit was seen when patients received everolimus exemestane. Fifty-two percent of patients had prolonged TTNT (> 6 months) with everolimus exemestane second-line and 45% had prolonged TTNT with everolimus exemestane third-line after prior endocrine therapy alone. In addition, 30% of patients had prolonged TTNT with everolimus exemestane second-line and 38% with everolimus exemestane third-line following prior endocrine therapy + CDK4/6i. This data suggests that everolimus exemestane may be an effective treatment regardless of prior line of therapy used.

There are several strengths and limitations to this study. This data included a large sample size that spans multiple geographic locations and different practice settings, and represents contemporary use of these therapies in the real-world setting. However, our analysis was limited by the lack of information regarding side effects, adverse events, adherence, dosage, or reasons for stopping or switching therapy. In addition, information regarding comorbidities is limited due to reliance on ICD codes which may not accurately capture all comorbidities. In addition, since these medications are oral, it can be difficult to assess exact treatment start and end dates or dose reductions when they are not recorded in the electronic health record. In Flatiron’s source database, abstraction of EHR information is used to confirm the documented therapy start date and last documented data of therapy.

The approval of CDK4/6 inhibitors in 2015 led to a novel first-line therapy option for hormone receptor-positive metastatic breast cancer patients and changed the algorithm of treatment options for these patients. Our findings suggest that among women with hormone-positive HER2− metastatic breast cancer, the use of everolimus exemestane after the development of CDK4/6 inhibitor resistance is still an effective treatment option and may delay the need for chemotherapy in this patient population.

Clinical trials are ongoing to assess the efficacy and safety of everolimus in combination with other therapies. Everolimus in combination with fulvestrant improved PFS to 10.3 months compared to 5.1 months with fulvestrant alone in patients with metastatic breast cancer resistant to aromatase inhibitor therapy [20]. A phase I/II trial of everolimus exemestane in combination with ribociclib reported a median PFS of 5.6 months, 8.4 months in the dose escalation group, and 12.7 months in the CDK4/6i naïve group, in patients with advanced hormone-positive breast cancer [21]. In addition, in May 2019, alpelisib in combination with fulvestrant was approved for PIK3CA mutated hormone-positive metastatic breast cancer based on the SOLAR-1 trial which reported PFS of 11 months [22]. Everolimus inhibits mTORC1 downstream of PI3K and may remain a valuable treatment option for patients who do not qualify for or who progress on alpelisib [23]. As new treatment combinations become available, more clinical trials will be needed to assess the optimal sequence of therapies.

## Conclusions

This study suggests that everolimus exemestane remains an effective treatment option after prior endocrine therapy or endocrine therapy in combination with CDK4/6 inhibitors. This real-word data shows an overall survival benefit when everolimus exemestane is used in the third-line setting following prior endocrine therapy with CDK4/6 inhibitors. The analysis suggests that this overall survival benefit is mainly driven by CDK4/6 inhibitors. As new treatment combinations become available, everolimus remains an option for patients who wish to delay chemotherapy.

## Supplementary Information

**Additional file 1 **: **Supplementary Table 1**. Therapy regimens that comprised the four treatment groups: everolimus exemestane, endocrine therapy, endocrine therapy + CDK 4/6i, and chemotherapy. **Supplementary Table 2.** Hazard ratios for multivariate analysis of survival among patients with advanced breast cancer by second- versus third-line everolimus exemestane treatment receipt. **Supplementary Figure 1.** a) Median time to next treatment for second-line everolimus exemestane treatment, stratified by prior treatment. b) Median time to next treatment for third-line everolimus exemestane treatment, stratified by prior treatment.

## Data Availability

All data generated or analyzed during this study are included in this published article and its supplementary information files.

## References

[1] DeSantis CE (2019). Breast cancer statistics, 2019. CA Cancer J Clin.

[2] Howlader N (2014). US incidence of breast cancer subtypes defined by joint hormone receptor and HER2 status. J Natl Cancer Inst.

[3] Finn RS (2015). The cyclin-dependent kinase 4/6 inhibitor palbociclib in combination with letrozole versus letrozole alone as first-line treatment of oestrogen receptor-positive, HER2-negative, advanced breast cancer (PALOMA-1/TRIO-18): a randomised phase 2 study. Lancet Oncol.

[4] Rugo HS (2018). Impact of palbociclib plus letrozole on patient-reported health-related quality of life: results from the PALOMA-2 trial. Ann Oncol.

[5] Rugo HS (2019). Palbociclib plus letrozole as first-line therapy in estrogen receptor-positive/human epidermal growth factor receptor 2-negative advanced breast cancer with extended follow-up. Breast Cancer Res Treat.

[6] Janni W (2018). First-line ribociclib plus letrozole in postmenopausal women with HR+, HER2- advanced breast cancer: Tumor response and pain reduction in the phase 3 MONALEESA-2 trial. Breast Cancer Res Treat.

[7] Goetz MP (2017). MONARCH 3: abemaciclib as initial therapy for advanced breast cancer. J Clin Oncol.

[8] Turner NC (2015). Palbociclib in hormone-receptor-positive advanced breast cancer. N Engl J Med.

[9] Malorni L (2018). Palbociclib as single agent or in combination with the endocrine therapy received before disease progression for estrogen receptor-positive, HER2-negative metastatic breast cancer: TREnd trial. Ann Oncol.

[10] Slamon, D. J. *et al.* Overall survival with ribociclib plus fulvestrant in advanced breast cancer. N. Engl. J. Med*.* 2019; NEJMoa1911149. doi:10.1056/NEJMoa1911149 10.1056/NEJMoa1911149 31826360

[11] Ribociclib as first-line therapy for HR-positive, advanced breast cancer. N. Engl. J. Med*.* 2018; 379:2582–2582. 10.1056/NEJMx180043 30586508

[12] Sledge G (2020). The effect of abemaciclib plus fulvestrant on overall survival in hormone receptor positive ERBB2 negative breast cancer that progressed on endocrine therapy-MONARCH 2. JAMA Oncol.

[13] Yardley DA (2013). Everolimus plus exemestane in postmenopausal patients with HR(+) breast cancer: BOLERO-2 final progression-free survival analysis. Adv Ther.

[14] Tesch H, et al. 4EVER-final efficacy analysis of the phase IIIb, multi-center, open label study for postmenopausal women with estrogen receptor positive locally advanced or metastatic breast cancer (BC) treated with everolimus (EVE) in combination with exemestane (EXE). Cancer Res 2015;75(9 Suppl). Abstract PS-19-06.

[15] Fasching PA, et al. Breast cancer treatment with everolimus and exemestane for ER+ women results of the 2^nd^ interim analysis of the non-interventional trial BRAWO. Ann Oncol 2014;4;25(4 Suppl) Abstract LBA9.

[16] Steger G, et al. Efficacy and safety of everolimus plus exemestane in HR+HER2- advanced breast cancer progressing on/after prior endocrine therapy in routine clinical practice: second interim analysis from STEPAUT. Cancer Res 2017;77(4 Suppl) Abstract P4–22-20.

[17] Curtis MD, Griffith SD, Tucker M, Taylor MD, Capra WB, Carrigan G, Holzman B, Torres AZ, You P, Arnieri B, Abernethy AP (2018). Development and validation of a high-quality composite real-world mortality endpoint. Health Serv Res.

[18] Elixhauser A, Steiner C, Harris DR, Coffey RM (1998). Comorbidity measures for use with administrative data. Med Care.

[19] Royce M (2018). Everolimus plus endocrine therapy for postmenopausal women with estrogen receptor-positive, human epidermal growth factor receptor 2-negative advanced breast cancer: a clinical trial. JAMA Oncol.

[20] Kornblum N (2018). Randomized phase II trial of fulvestrant plus everolimus or placebo in postmenopausal women with hormone receptor positive HER2 negative metastatic breast cancer resistant to aromatase inhibitor therapy: results of PrE0102. J Clin Onc.

[21] Bardia A, et al. Phase Ib dose-escalation/expansion trial of ribociclib in combination with everolimus and exemestane in postmenopausal women with HR^+^, HER2^−^ advanced breast cancer. Clin Cancer Res. 2020;26(24):6417–28. 10.1158/1078-0432.CCR-20-1068 PMC8489181 32998962

[22] Andre F (2019). Alpelisib for PIK3CA-mutated hormone receptor positive advanced breast cancer. N Engl J Med.

[23] Vernieri C (2020). Everolimus versus alpelisib in advanced hormone receptor positive HER2 negative breast cancer: targeting different nodes of the PI3K/AKT/mTORC1 pathway with different clinical implications. Breast Cancer Res.

