# Supplementary material for: Patterns of treatment with everolimus exemestane in hormone receptor-positive HER2-negative metastatic breast cancer in the era of targeted therapy
Source: Breast Cancer Res. 2021 Jan 29;23:14. doi: 10.1186/s13058-021-01394-y (PMC7844919; doi:10.1186/s13058-021-01394-y)
Supplement: Supplementary file 1 — Additional file 1 : Supplementary Table 1. Therapy regimens that comprised the four treatment groups: everolimus exemestane, endocrine therapy, endocrine therapy + CDK 4/6i, and chemotherapy. Supplementary Table 2. Hazard ratios for multivariate analysis of survival among patients with advanced breast cancer by second- versus third-line everolimus exemestane treatment receipt. Supplementary Figure 1. a) Median time to next treatment for second-line everolimus exemestane treatment, stratified by prior treatment. b) Median time to next treatment for third-line everolimus exemestane treatment, stratified by prior treatment. [file 13058_2021_1394_MOESM1_ESM.docx]

**Supplementary Table 1.** Therapy regimens that comprised the four treatment groups: everolimus exemestane, endocrine therapy, endocrine therapy + CDK 4/6i, and chemotherapy.

| **Treatment group** | **Regimens** |
| --- | --- |
| Everolimus and Exemestane (EE) | everolimus, exemestane +/- leuprolide or goserelin |
| Endocrine therapy alone (ET alone) | Anastrozole  Exemestane  Fulvestrant  Letrozole  Tamoxifen  +/- leuprolide or goserelin |
| CDK4/6i + ET | Any aromatase inhibitor, abemaciclib +/- leuprolide or goserelin  Any aromatase inhibitor, ribociclib +/- leuprolide or goserelin  Any aromatase inhibitor, palbociclib +/- leuprolide or goserelin  Fulvestrant, abemaciclib +/- leuprolide or goserelin  Fulvestrant, ribociclib +/- leuprolide or goserelin  Fulvestrant, palbociclib +/- leuprolide or goserelin |
| Chemotherapy | capecitabine, carboplatin, cisplatin, cyclophosphamide, docetaxel, doxorubicin, eribulin, fluorouracil, gemcitabine, irinotecan, paclitaxel, vinorelbine (any of the above alone or in combination) |

**Supplementary Table 2**. Hazard ratios for multivariate analysis of survival among patients with advanced breast cancer by second- versus third-line everolimus exemestane treatment receipt.

| **Characteristic** | **Everolimus and Exemestane, Second-Line** | | **Everolimus and Exemestane, Third-Line** | |
| --- | --- | --- | --- | --- |
|  | **HR (95% CI)** | ***p*** | **HR (95% CI)** | ***p*** |
| **Age** |  |  |  |  |
| < 60 years | 1 (ref) | -- | -- | -- |
| ≥ 60 years | 0.54 (0.34, 0.86) | 0.010 | -- | -- |
| **Insurance** |  |  |  |  |
| Medicare | 1 (ref) | -- | -- | -- |
| Commercial | 0.49 (0.28, 0.86) | 0.013 | -- | -- |
| Other, including Medicaid | 0.47 (0.21, 1.01) | 0.071 | -- | -- |
| **Site of Metastasis** |  |  |  |  |
| Visceral | -- | -- | 1 (ref) | -- |
| Non-Visceral | -- | -- | 0.49 (0.27, 0.91) | 0.024 |
| **Elixhauser Comorbidity Index Score** |  |  |  |  |
| 0 | -- | -- | 1 (ref) | -- |
| 1 | -- | -- | 3.06 (1.29, 7.27) | 0.012 |
| 2+ | -- | -- | 0.55 (0.08, 3.96) | 0.549 |
| **ECOG** |  |  |  |  |
| 0 | -- | -- | 1 (ref) | -- |
| 1 | -- | -- | 1.29 (0.73, 2.30) | 0.378 |
| 2 | -- | -- | 4.60 (1.71, 12.43) | 0.003 |
| 3 | -- | -- | 2.14 (0.50, 9.14) | 0.305 |
|  |  |  |  |  |

**Supplementary Figure 1**. a) Median time to next treatment for second-line everolimus exemestane treatment, stratified by prior treatment. b) Median time to next treatment for third-line everolimus exemestane treatment, stratified by prior treatment.

a)
b)
